# Bayesian Analyses of Comparative Data with the Ornstein–Uhlenbeck Model: Potential Pitfalls

**DOI:** 10.1093/sysbio/syac036

**Published:** 2022-05-18

**Authors:** Josselin Cornuault

**Affiliations:** Real Jardín Botánico (RJB), CSIC, Plaza de Murillo, 28014 Madrid, Spain; ISEM, Université de Montpellier, CNRS, IRD, EPHE, Place Eugène Bataillon, 34095 Montpellier, France

## Abstract

The Ornstein–Uhlenbeck (OU) model is widely used in comparative phylogenetic analyses to study the evolution of quantitative traits. It has been applied to various purposes, including the estimation of the strength of selection or ancestral traits, inferring the existence of several selective regimes, or accounting for phylogenetic correlation in regression analyses. Most programs implementing statistical inference under the OU model have resorted to maximum-likelihood (ML) inference until the recent advent of Bayesian methods. A series of issues have been noted for ML inference using the OU model, including parameter nonidentifiability. How these problems translate to a Bayesian framework has not been studied much to date and is the focus of the present article. In particular, I aim to assess the impact of the choice of priors on parameter estimates. I show that complex interactions between parameters may cause the priors for virtually all parameters to impact inference in sometimes unexpected ways, whatever the purpose of inference. I specifically draw attention to the difficulty of setting the prior for the selection strength parameter, a task to be undertaken with much caution. I particularly address investigators who do not have precise prior information, by highlighting the fact that the effect of the prior for one parameter is often only visible through its impact on the estimate of another parameter. Finally, I propose a new parameterization of the OU model that can be helpful when prior information about the parameters is not available. [Bayesian inference; Brownian motion; Ornstein–Uhlenbeck model; phenotypic evolution; phylogenetic comparative methods; prior distribution; quantitative trait evolution.]

Phylogenetic comparative methods (PCMs) aim to analyze data sets of species traits in a phylogenetic framework. They are used for various purposes, including detecting selection (e.g., Martins 1994; Butler and King 2004; Ingram and Mahler 2013), measuring the rate of trait evolution (e.g., O’Meara et al. 2006; Revell and Harmon 2008; Revell and Collar 2009; Eastman et al. 2011; Jones et al. 2015; Martin 2016), estimating ancestral traits (e.g., Martins and Hansen 1997; Bokma 2002; Paradis et al. 2004; Harmon et al. 2008; Lemey et al. 2010; Revell 2012; Landis et al. 2013; Elliot and Mooers 2014; Kratsch and McHardy 2014; Meseguer et al. 2018), control for the phylogenetic codependency of multispecies data (e.g., Felsenstein 1985; Grafen 1989), or estimate the impact of a covariate on a trait (e.g., Davis et al. 2012; Gohli and Voje 2016; Solbakken et al. 2017; Lattenkamp et al. 2021). 

The first model to be considered in PCMs was Brownian motion (BM) (Cavalli-Sforza and Edwards 1967; Felsenstein 1973), a model assuming that traits evolve in an undirected manner, at a speed governed by a rate parameter. Then, the Ornstein–Uhlenbeck (OU) model has been used as a model of stabilizing selection and drift (Lande 1976,Hansen and Martins 1996,Felsenstein 1988). Under the OU model, traits are deterministically attracted towards a selective optimum, at a speed determined by a selection strength parameter. Stochastic variation around this selection-driven trajectory follows a Brownian model (Hansen and Martins 1996). BM is therefore a limiting case of the OU model when there is no selection. A notable difference between the OU and Brownian models is that under the OU model, the imprint of shared evolutionary history on species traits is progressively erased due to the convergent effect of selection (Hansen and Martins 1996). 

The Brownian and OU models have burgeoned into a number of extensions, including models with heterogeneous evolutionary rates (e.g., O’Meara et al. 2006; Lemey et al. 2010; Eastman et al. 2011), early-burst models of adapative radiation (Harmon et al. 2010) (see also Blomberg et al. 2003; Freckleton and Harvey 2006), models of punctuated evolution (e.g., Bokma 2002; Uyeda et al. 2011; Landis et al. 2013; Elliot and Mooers 2014), or multivariate models (e.g., Hansen and Martins 1996; Revell and Harmon 2008; Revell and Collar 2009). Also, recognizing that selection is a dynamic process, the OU model has been extended to accommodate multiple selective optima. This model, known as the Hansen model (Hansen 1997), assumes various selective regimes across the phylogeny. The Hansen model has been used for testing a priori evolutionary hypotheses characterized by specific “paintings” of selective regimes on the tree (e.g., Hansen 1997; Butler and King 2004), or for estimating the location of selective regimes on the tree (e.g., Ingram and Mahler 2013; Uyeda and Harmon 2014). 

A number of issues have been reported in maximum-likelihood (ML) inference with the OU model (see notably Ané 2008,Boettiger et al. 2012,Ho and Ané 2013, 2014; Cooper et al. 2016). In particular, Ho and Ané (2014) reported that the selective optimum and the ancestral trait at the root of the tree are not separately identifiable (i.e., infinitely many pairs of values for these parameters have the same likelihood, impeding their joint estimation). This situation generalizes, under some conditions, to the Hansen model with multiple selective optima. Also, when the estimated value of selection strength is close to 0, the location of the selective optimum or the number of selective regimes cannot be estimated (Ho and Ané 2014). Ho and Ané (2014) stress that these issues should not be restricted to ML inference and also concern Bayesian inference. 

In recent years, a number of programs have arisen that extend statistical comparative biology to Bayesian inference, in particular, the R packages bayou (Uyeda and Harmon 2014), POUMM (Mitov and Stadler 2017), and the program RevBayes (Höhna et al. 2016). The models used in Bayesian inference are characterized by the same probabilistic models as their ML analogs, but notably differ in their ability to include subjective prior information. There is thus the potential for the prior to have a strong influence on the results in case the data are not very informative, or in case parameters are not separately identifiable, as is the case for the OU model (Ho and Ané 2013, 2014). Although it has been noticed that the prior for the number of selective regimes in the Hansen model could strongly influence the results (Uyeda and Harmon 2014), to this day, little is known about the impact of the prior when fitting the OU model. 

This article studies how some of the problems identified in ML inference with the OU and Hansen models manifest themselves in Bayesian inference. Of particular interest, are the potential consequences of the nonidentifiability (or weak identifiability) of some parameters (the selective optimum and the initial state at the root on the one hand, and the selection strength and the rate of evolution on the other hand) in the case where prior information is vague. Nonidentifiability is shown to induce correlation among parameters in the posterior, so that the prior for one parameter may strongly impact the posterior of another. It is important, in that case, that investigators are aware that part of the difference between the posterior and the prior of a parameter is due to the prior of another parameter. Furthermore, the parameter for the strength of selection ($\alpha$) plays a central role, as it interacts with all other parameters. All conclusions are therefore conditional on the correct estimation of $\alpha$, and the prior for $\alpha$ should be set with the greatest care. I emphasize that, in the absence of precise prior information about $\alpha$ and/or the rate of evolution, setting this prior is not straightforward. A reparameterization of the OU model in terms of only identifiable parameters is proposed, which I hope makes it easier to set priors when no prior information is available. 

## Overview of Models

### OU Model

Consider a rooted ultrametric tree $T$ and a set of observed tip traits $\mathbf{Y}$ (one value per species). Under the OU model, $\mathbf{Y}$ is multivariate normally distributed, with the same expected trait value for all species:

(1) \begin{equation*} M_{\rm OU}=\theta\left(1-e^{-\alpha t_{H}}\right)+Y_{r}e^{-\alpha t_{H}},\end{equation*}

where $\theta$ (in unit of traits) is the selective optimum, $Y_{r}$ is the root trait, $\alpha$ is the strength of selection (in unit of $\text{time}^{-1}$), and $t_H$ (in unit of time) is the tree height. Thus, under the OU model, expected trait values are deterministically attracted from the initial value $Y_{r}$ to the optimum $\theta$ as time passes. The stronger the selection, the faster the trait values are expected to approach $\theta$.

The variance–covariance structure of the OU distribution is given by the matrix:

(2) \begin{equation*} \Sigma_{\rm OU}=\sigma^{2} \mathbf{C}(T,\alpha),\end{equation*}

where the stochastic rate $\sigma^{2}$ (in unit of squared trait/time) scales the amount of stochasticity about $M_{\rm OU}$. The matrix $\mathbf{C}(T,\alpha)$ determines the expected covariation between the trait values of the different species, given the phylogeny and $\alpha$ (see Hansen 1997, and Appendix 1 of the Supplementary material available on Dryad at https://doi.org/10.5061/dryad.vdncjsxrc for details). In particular, $\mathbf{C}(T,\alpha)$ assumes a certain degree of phylogenetic signal, that is, the more recent the tMRCA of two species, the more similar their trait values are expected to be. The value of $\alpha$ then determines how quickly the phylogenetic signal is erased by the homogenizing effect of selection: the higher $\alpha$, the quicker the phylogenetic signal is lost.

We now define two limiting forms of the OU model: BM, which is the OU model with $\alpha\rightarrow0$, and the white-noise (WN) model, with $\alpha\rightarrow+\infty$ (see Cooper et al. 2016). 

Under BM, without selection, the expected trait value of all current species given $Y_r$ stays constant as time passes: $M_{\rm OU}=Y_r$. Also, the phylogenetic signal of traits reaches a maximum as compared to higher values of $\alpha$. 

The other extreme is the WN model, with mean $M_{\rm OU}=\theta$: selection is so fast that all trait values are expected to be almost immediately located around the optimum. The phylogenetic signal gets down to zero, meaning that the phylogenetic relationships among species no longer influence the trait values. The trait value of a species is thus independent from the trait values of the other species and varies around $\theta$ with variance $\eta=\frac{\sigma^{2}}{2\alpha}$ (see Appendix 1 of the Supplementary material available on Dryad). 

See Appendix 1 of the Supplementary material available on Dryad for the expression of $\mathbf{C}(T,\alpha)$ and more details on the OU model. 

### Hansen Model

Hansen’s model (Hansen 1997) is identical to the OU model, except that $\theta$ is allowed to vary among branches, which changes the mean of trait $Y_i$ of species $i$ into:

$$
M_{Hi}=Y_{r}e^{-\alpha t_H}+ k_i,$$

where $k_i$ is a function of $\alpha$, $T$, and the set of optima along the path in the tree from the root to species $i$.

As for the OU model, in the limit of $\alpha\rightarrow0$, the Hansen model converges to BM, with $M_{Hi}=Y_r$ for all $i$. In the limit of $\alpha\rightarrow+\infty$, it converges to a WN model with different means for the different regimes: $M_{Hi}=\theta_i$, with $\theta_i$ the optimum for species $i$. The variance–covariance structure of the Hansen model is identical to the OU model, with the same implications about the impact of $\alpha$ on the amount of phylogenetic signal. See Appendix 1 of the Supplementary material available on Dryad for the expression of $k_i$ and more details on Hansen’s model. 

## Measuring the Realized Effect of Selection

In the previous section, we have seen how the BM and WN models are limiting forms of the OU model that arise as $\alpha\rightarrow0$ or $\alpha\rightarrow+\infty$, respectively. However, these models are only extremes of a continuum along which the importance of selection in determining the observed trait values increases. 

Throughout this article, I will use the metric $\rho$ (slightly modified from Hansen et al. 2008) as a measure of the realized effect of selection in determining observed trait values, for a given OU process:

(3) \begin{equation*} \begin{split} \rho(t_H) &= 1 - \frac{V_{\rm OU}(t_H)}{V_{\rm BM}(t_H)} = 1 - \frac{1-\exp (-2\alpha t_H)}{2 \alpha t_H},\end{split} \end{equation*}

where $V_{\rm OU}(t_H)$ is the expected variance of the OU process at the time of sampling (i.e., at time $t_H$ above the root) and where $V_{\rm BM}(t_H) \geq V_{\rm OU}(t_H)$ is the variance that would be expected under BM (i.e., if $\alpha$ was 0). See Appendix 2 of the Supplementary material available on Dryad for details.

The metric $\rho$ thus represents the percent decrease in trait variance caused by selection over the study period ($t_H$), as compared to the variance expected under pure drift (i.e., under BM). For instance, $\rho = 0.25$ means that selection has reduced the variance of traits by 25$\%$ over period $t_H$. 

**Figure 1:**
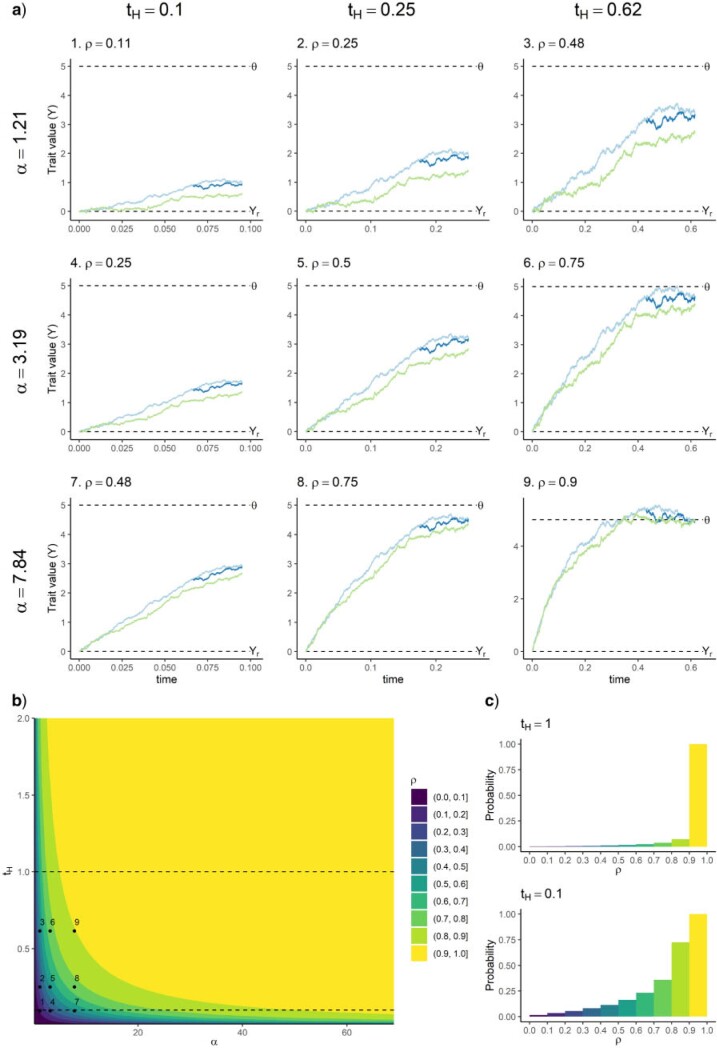
a) Trait trajectories for a three-tip tree for nine different combinations of values of $\alpha$ and $t_H$. b) Value of $\rho$ as a function of $\alpha$ and $t_H$. The nine points correspond to the nine trajectories in (a). Note that the analysis of a given data set with a given $t_H$ occurs along a horizontal line of this graph. c) Priors for $\rho$ corresponding to a uniform prior for $\alpha$ from 0 to 69, when $t_H=1$ or $t_H=0.1$. The relative heights of the different bars match the relative distances between the $\rho$ isoclines in b), along the lines $t_H=1$ or $t_H=0.1$ (represented by dashed lines in b).

Notice that the fact that $\rho$ depends only on the product $\alpha t_H$ implies that it measures the realized effect of selection (Cressler et al. (2015) called $\alpha t_H$ the “opportunity for selection”). In a case where $\alpha$ is high, but the process has run for a very small period of time (very low $t_H$), $\rho$ will be low, reflecting the fact that selection (although it is strong) has not had time to substantially influence the evolution of traits. Conversely, $\alpha$ may be low and $t_H$ very high, in which case $\rho$ is high because the accumulation of slow selection over a large period of time did influence substantially the evolution of traits. Thus, $\rho$ describes the net macroevolutionary effect produced by the accumulation of microevolutionary selective effects (the magnitude of which is described by $\alpha$), over a given period of time ($t_H$). Figure 1 shows the relationship between $\rho$, $\alpha,$ and $t_H$ and illustrates how the value of $\rho$ reflects the shape of trait evolutionary trajectories. Further notice that $\rho$ is a direct indicator of how close an OU model actually is from the BM and WN models, statistically, with a high (respectively low) value of $\rho$ indicating closeness to a WN (respectively BM) model (see Appendix 2 of the Supplementary material available on Dryad for details). 

In sum, $\rho$ measures the realized impact of selection, given the length $t_H$ that the process has run, while $\alpha$ measures the absolute strength of selection, independently of $t_H$. 

Another interesting transform of $\alpha$ is the phylogenetic half-life $t_{1/2}=\ln 2 / \alpha$, which represents the expected time needed for a trait to cover half the distance between the initial value $Y_r$, and the selective optimum $\theta$. Phylogenetic half-life, like $\alpha$, measures the absolute magnitude of selection but is probably easier to interpret. Some authors have preferred to scale $t_{1/2}$ in units of tree heights (e.g., Hansen et al. 2008; Ané et al. 2017; Cooper et al. 2016), in which case, like $\rho$, it depends on the product $\alpha t_H$ and is then a measure of the realized effect of selection over time $t_H$. 

I elaborate in the discussion on the pros and cons of interpreting $\alpha$, $t_{1/2}$, or $\rho$. Meanwhile, I use $\rho$ to interpret the value of $\alpha$ in terms of the realized impact of selection, given some $t_H$. In particular, this article aims to illustrate how the prior assigned to $\alpha$ may translate into a very stringent prior for $\rho$, in favor of either BM (low $\rho$) or WN (high $\rho$) processes, and how such stringent priors may impact the estimation of the other parameters, as a result of complex interactions among parameters. 

## Interactions among the Parameters of the OU Model

The probability distribution of trait values (i.e., the likelihood) under the OU model is characterized by two ridges. The occurrence of a ridge in a likelihood function implies that the data are equally likely for infinitely numerous combinations of values of a set of parameters, implying that the values of the parameters cannot be separately identified. 

**Figure 2:**
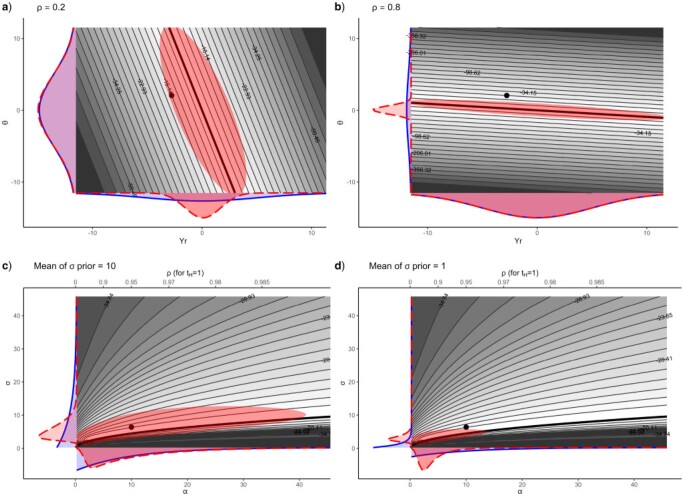
Example ridges in the OU likelihood. Gray shades represent the value of the log-likelihood, for a data set simulated with $\alpha=0.69$, of which the trait values were scaled to mean 0 and unit variance. Density plots with solid lines in the margins represent the priors. The darker areas superimposed on the likelihood surface represent the 95$\%$ highest posterior density region of the joint posterior of $\theta$ and $Y_r$ (in a and b) or of $\sigma$ and $\alpha$ (in c and d). Density plots with dashed lines in the margins represent the marginal posteriors. The posteriors were approximated by numerical integration. Black dots represent the true values of the parameters used to simulate the data set. a) and b) represent the $\theta-Y_r$ ridge, with $\sigma$ fixed to its true value and with $\rho=0.2$ (a) or $\rho=0.8$ (b). In both cases, the priors for $\theta$ and $Y_r$ are centered normal distributions with sd = 5. The thick black line is the top of the ridge, of equation $\theta (1-\exp(-\alpha t_H)) + Y_r \exp(-\alpha t_H) = \hat{M}_{\rm OU}$, with $\hat{M}_{\rm OU}$ the ML estimator of the mean of tip trait values (equal to 0 in this example, since trait values were scaled to 0 mean). In a), because $\rho$ is low, the ridge has a highly negative slope. As a consequence, the plausible range of $\theta$, as constrained by the $\theta$ prior, corresponds to a narrow interval of high likelihood on the scale of $Y_r$, inducing a marginal posterior for $Y_r$ that is narrower than its prior. In b), $\rho$ is high and the converse happens. c) and d) The $\sigma-\alpha$ ridge, with $\theta$ and $Y_r$ fixed to their true values is represented. The prior for $\alpha$ is in both cases an exponential distribution with mean 10. The prior for $\sigma$ is an exponential distribution with mean 10 in c) and with mean 1 in d). The thick black line has equation $\eta=\sigma^2/(2\alpha)$, with $\eta$ the stationary variance of the process, set to 1 (the sample variance of the tip trait values after scaling). As $\alpha$ grows, this line tends to be the top of the ridge. The difference with the $\theta-Y_r$ ridge is that the top of this ridge is not completely flat. However, as one moves towards higher $\alpha$ values, it gets ever flatter. In d), the prior for $\sigma$ restricts inference to smaller values of $\sigma$ than in c). As a consequence, the a priori smaller plausible values of $\sigma$ correspond to a region around the likelihood ridge that matches smaller $\alpha$ values. This has the effect of shifting the marginal posterior of $\alpha$ towards smaller values. In this example, the true value of $\alpha$ would even be excluded from the 95$\%$ credible interval, because of the prior for $\sigma$.

This happens the first time for the parameters $\theta$ and $Y_{r}$, which cannot be separately identified under the OU model when only contemporaneous tip trait values are observed (Ho and Ané 2014). Specifically, when tips are contemporaneous, the OU likelihood is invariant along the following ridge in the $\theta - Y_r$ plane (see Fig. 2a,b):

$$
\theta (1-\exp(-\alpha t_H))+Y_{r}\exp(-\alpha t_H)=M_{\rm OU},$$

where $M_{\rm OU}$ is any value considered for the expected value of tip traits. Indeed, there are infinitely numerous pairs of values of $\theta$ and $Y_{r}$ that induce the same value of $M_{\rm OU}$ (hence not changing the value of the likelihood), for a given value of $\alpha t_H$ (hence for a given value of $\rho$).

Nonidentifiability of parameters can be partially remedied by resorting to Bayesian inference, which allows including additional information about parameter values by specifying prior distributions, thus limiting the range of plausible values for parameters. For instance, specifying priors for $Y_{r}$ and $\theta$ will constrain inference to remain within a given, plausible part of the ridge represented in Figure 2. Figure 2a shows that, when $\rho$ is low, constraining the range of $\theta$ by way of a prior may constrain the posterior range of $Y_{r}$. In this case, the investigator will rightfully deduce that the posterior of $Y_{r}$ is narrower than its prior because the data carries information about $Y_{r}$. However, this conclusion is conditional on: 1) $\alpha$ (equivalently $\rho$) being correctly estimated and 2) the prior for $\theta$. Indeed, if $\rho$ is estimated to be high (e.g., because of the prior for $\alpha$, see next section), the estimated shape of the ridge would be more like that of Figure 2b, in which case it is the prior for $Y_{r}$ that constrains the width of the posterior of $\theta$. Also, if one had chosen a wider prior for $Y_r$, the posterior of $\theta$ would have been wider. This shows how the nonidentifiability of $Y_{r}$ and $\theta$ under the OU model makes the conclusions of Bayesian inference highly dependent on the relative widths of the prior distributions of $Y_{r}$ and $\theta$, and on the estimate of $\alpha$.

A second ridge occurs in the $\sigma - \alpha$ plane. The difference with the $\theta - Y_r$ ridge is that the top of the $\sigma - \alpha$ ridge is not completely flat: it seems that there is always one single pair of values of $\sigma$ and $\alpha$ that maximizes the likelihood, for any values of the other parameters ($\theta$ and $Y_r$). However, as $\alpha$ increases, the top of the ridge becomes increasingly flat, and $\sigma$ and $\alpha$ become less and less separately identifiable. Indeed, we have seen above that as $\alpha$ increases, the OU likelihood progressively becomes similar to the WN likelihood, where the expected value of tip traits is $M_{OU}=\theta$, and the variance of tip traits is $\eta = \sigma^2 / (2 \alpha)$. Thus, if $\alpha$ is estimated to be sufficiently high that the WN model can be substituted for the OU model, any two pairs of values of $\sigma$ and $\alpha$ yielding the same value of $\eta$ are almost equally likely. This is visible in Figure 2c,d, where we observe a ridge in the $\sigma - \alpha$ plane, of equation:

$$
\sigma^2 =2 \alpha \eta.
$$

The existence of this second ridge implies that the estimates of $\alpha$ and $\sigma$ may be correlated, which provides grounds for the priors of one of these parameters to impact the estimate of the other parameter. For instance, Figure 2c,d shows the posteriors of $\sigma$ and $\alpha$ obtained with a wider prior for $\sigma$ (with mean 10) and for a narrower prior (with mean 1). We can see that the narrower prior for $\sigma$ induces a narrower posterior, not only for $\sigma$ but also for $\alpha$. This emphasizes that the choice of prior for $\sigma$ may impact the estimate of $\alpha$ or vice versa. 

In summary, the mathematical structure of the OU model is such that the estimates of $\sigma$, $\theta,$ and $Y_r$ are highly conditional on the value of $\alpha$, and on their priors. If the data are very informative, the estimate of $\alpha$ will depend little on the priors. But if the data are not so informative, or if some very stringent priors are used, the outcome of the analysis will be very dependent on the priors. 

## Setting a Prior for the Selection Strength

In Bayesian inference, investigators must specify prior distributions for all parameters. With the OU or Hansen models, the choice of prior for $\alpha$ is very important, as it may influence the estimation of all other parameters (as detailed in the previous section), yet this is anything but an intuitive task. 

When an investigator undertakes the task to set a prior, they may be in one of two cases: 1) they have rather precise biological information about the value of the parameter, 2) they have rather vague information, or no information at all. The first situation is less prone to errors: the investigator will choose a narrow prior which covers the a priori credible zone adequately. In the second situation, the investigator mainly wants the prior to reflect their large uncertainty about the parameter, which is not as straightforward as it seems. 

To do so, the investigator’s first instinct may be to use a flat prior that covers all the plausible values of the parameter, for any natural system similar to that under study. It is even often the case that the investigator extends the range of the prior far beyond plausible values, confident that the flatness of the prior guarantees that the prior will have no effect. This was my first instinct when I first came into contact with the OU model and I set an exponential prior for $\alpha$ with a very large mean. 

As an example of a flat prior for $\alpha$, let us consider a uniform prior distribution:

$$
\alpha\sim\text{Uniform}\left(0,c\right.).
$$

Let us assume branch lengths in our phylogeny are in Ma, so that $\alpha$ is in Ma$^{-1}$. We want to decide on an upper bound $c$ so that $\alpha\in\left[0,c\right]$ covers the whole range of plausible values of $\alpha$ that we may expect in nature. It is hard to interpret biologically the value of $\alpha$, so let us consider instead the phylogenetic half-time $t_{1/2}=\ln 2 / \alpha$ (see Hansen 1997; Cooper et al. 2016), which represents the time necessary for the process to cover half the way between $Y_r$ and $\theta$. We are looking for a small value $t_{1/2}=\ln2/c$ that represents strong-enough selection. By browsing the literature, we realize that there certainly are cases (for some specific traits and organisms) where rapid selection has been shown to change significantly the value of a trait over, say, 10,000 years. So let us choose $t_{1/2}=0.01 $ Ma, so that $c=\ln2/0.01\approx69$ Ma$^{-1}$ represents a high-enough value of $\alpha$. 

Now, if we look at this prior on the scale of $\rho$ for trees of height $t_H=1$ or $t_H=0.1$ (see Fig. 1c), we see that in both cases the prior for $\rho$ is not flat at all and favors WN-like models a lot. The difference between the shape of the prior for the two tree heights further illustrates that the same prior for $\alpha$ for data sets with different $t_H$ translates into different priors for $\rho$: the smaller $t_H$ is, the more the prior favors small values of $\rho$ (i.e., more BM models). 

Also notice that, as shown in Appendix 3 of the Supplementary material available on Dryad, setting a flat prior on $t_{1/2}$ instead of $\alpha$ may well result in a very Brownian prior or very WN prior. 

**Figure 3:**
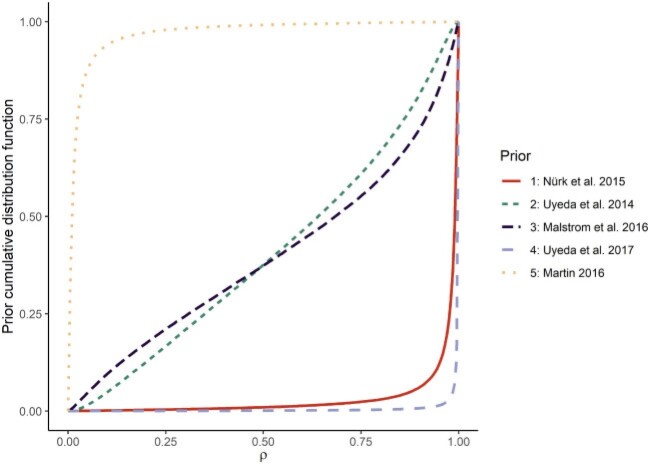
A set of five priors for $\alpha$ found in the literature, translated into priors for $\rho$. These priors can be observed to range from favoring a lot low values of $\rho$ (Martin 2016) or high values of $\rho$ (e.g., Uyeda et al. 2017).

In conclusion, the interpretation of a vague prior for the strength of selection highly depends on the chosen scale ($\alpha$, $t_{1/2}$, or $\rho$). We therefore need to decide whether we are more comfortable interpreting a prior for 1) selection’s absolute strength ($\alpha$), 2) the lag time for selection to have a certain effect ($t_{1/2}$), 3) the realized effect of selection in shaping trait values ($\rho$), or 4) yet another meaningful transform of $\alpha$. I discuss these options in the discussion, but for now, it is important to notice that if we do not choose a prior that blends relatively evenly the different values of $\rho$, we may end up with a prior that favors overwhelmingly one of the two extremes of the continuum: BM or WN processes. A nonexhaustive review of papers fitting the OU or Hansen models using Bayesian inference shows that the priors assigned to $\alpha$ range from very BM processes to very WN processes, corresponding to very different a priori assumptions about the role of selection in shaping trait values. These priors are represented on the scale of $\rho$ in Figure 3. The two most extreme priors are that of Martin (2016), very Brownian with 95$\%$ of the prior density below $\rho = 0.12$ (hereafter the BM prior) and that of Uyeda et al. (2017), very WN with 95$\%$ of the prior density above $\rho = 0.98$ (hereafter the WN prior). Note that these two studies used the same prior for $\alpha$ (a half-Cauchy distribution with scale 1), but for very different tree heights, which translates into very different priors for $\rho$. Martin (2016) analyzed a tree with 42 taxa (comparable to the simulations of this study) while Uyeda et al. (2017) studied a tree with 857 taxa, which is probably much less sensitive to the choice of prior. Note that the investigators of these studies have probably based their choice of prior on sensible considerations and I only want here to point out that a wide variety of priors are used, when looking on the scale of $\rho$. 

## Simulation Study

In order to investigate the impact of the choice of prior for $\alpha$, I simulated data sets under various processes and analyzed them using one or the other of the two extreme priors for $\alpha$: the BM prior of Martin (2016), and the WN prior of Uyeda et al. (2017). Notice that the goal of these analyses is to see the cascading effects of the $\alpha$ prior to the estimates of other parameters. To this aim, I deliberately choose the priors for the other parameters to be sufficiently wide around their known true value to show the effects of among-parameter correlations. It is understood that the priors of the present analysis are not meant to represent good practice. 

### Main Analytical Setting

Trees of height 1 were simulated with $n = 10$, 20, or 40 tips according to a Yule process with a speciation rate equal to $\ln(n)$. Twenty replicate trees were simulated for each value of $n$ (60 trees in total). Continuous traits were simulated along each tree according to three processes:

- Brownian motion, with $Y_{r}=0$ and $\sigma^{2}=1$
- a WN-like model with $\theta=1$ and $Y_{r}=0$, with $\alpha=5$ (i.e., $\rho\simeq0.90$ for a tree of height 1) and $\sigma^{2}=1$
- a Hansen model with two selective regimes, one for each of the two descendant lineages of the root, with $\theta_{1}=-1$ and $\theta_{2}=1$, $Y_{r}=0$, $\alpha=5,$ and $\sigma^{2}=1$. So that we do not end up with selection regimes applying to a low number of lineages, the simulated trees were conditioned on having at least 3, 5, and 10 tips on either side of the root for $n$ = 10, 20, and 40, respectively.

In total, 180 data sets were generated (3 tree sizes $\times$ 3 sets of trait values $\times$ 20 replicates). Simulated ancestral trait values at internal nodes were recorded along with tip trait values and the phylogeny. 

The OU and Hansen models were fitted to the simulated data sets with RevBayes (Höhna et al. 2016). The RevBayes code used for these analyses is a modification of RevBayes tutorials (May 2019a,b) and is available in Appendix 4 of the Supplementary material available on Dryad. Ancestral trait values for all nodes were estimated. 

Tip trait values were normalized to mean 0 and unit variance before inference, so that the same priors could be used for all analyses. The parameters in units of traits (i.e., $Y_r$ and $\theta$) estimated from the normalized data (scaled parameters) can be converted back to their original unit (unscaled parameters) if needed. Scaled parameters are useful for comparison with the prior, because the scaled prior was the same for all analyses. However, they cannot be compared to the true values of parameters used in simulation, since these were expressed in unscaled trait units (see Appendix 5 of the Supplementary material available on Dryad for detail). Figures show unscaled parameters, unless stated otherwise. The unit of $\alpha$ is $\text{time}^{-1}$ and is thus invariant to trait scaling. 

For each analysis, one Monte Carlo Markov Chain (MCMC) was run for 100,000 iterations after a burnin period of 10,000. A second run was made for 60 analyses, showing that different runs yielded qualitatively similar results. 

The effect of the prior for $\alpha$ on estimated parameters was studied through two series of analyses.

1. An OU model was fitted to each data set with the BM and the WN priors. This amounted to 360 analyses (180 data sets $\times$ 2 priors).
2. I proceeded as in 1., except the Hansen model was fitted instead of the OU model.

Details on the priors can be found in Appendix 3 of the Supplementary material available on Dryad, and the R code and files for running the simulations and prepare the RevBayes scripts are given in Appendix 4 of the Supplementary material available on Dryad. 

### Additional Analyses

I assessed the sensitivity of the results of the OU analyses (point 1 above) with trees of 40 tips to three aspects by rerunning these analyses changing one thing at a time. Ancestral trait values were not estimated. First, trees with 300 tips were simulated instead of 40 to investigate a case where the likelihood may be very informative and dominate the prior. Second, trees were simulated under a birth–death model instead of a Yule model, with a net diversification rate $\lambda-\mu=\ln40$ and a turnover rate $\mu/\lambda=0.9$. Trees generated in this way have shorter terminal branches than Yule trees, which may preserve phylogenetic signal for higher values of $\alpha$. The value of $\mu/\lambda$ has been shown to have substantial effects in ML parameter inference (Cooper et al. 2016). Third, a flatter prior was considered for $\sigma$ (with mean 100 instead of mean 10 in the main analyses), to determine if this could impact the estimation of $\alpha$, as suspected above (see Fig. 2). 

## Results

All the results shown in the main text are for trees with 40 tips. Results for trees with 10 or 20 tips are qualitatively similar and can be found in Appendix 6 of the Supplementary material available on Dryad, along with a full report on MCMC mixing. The results of the three additional analyses are given in Appendix 7 of the Supplementary material available on Dryad, and mentioned where appropriate in the main text. The posterior distributions for ancestral trait values are qualitatively similar to those of $Y_r$ and can be found in Appendix 8 of the Supplementary material available on Dryad. 

### OU Model

**Figure 4:**
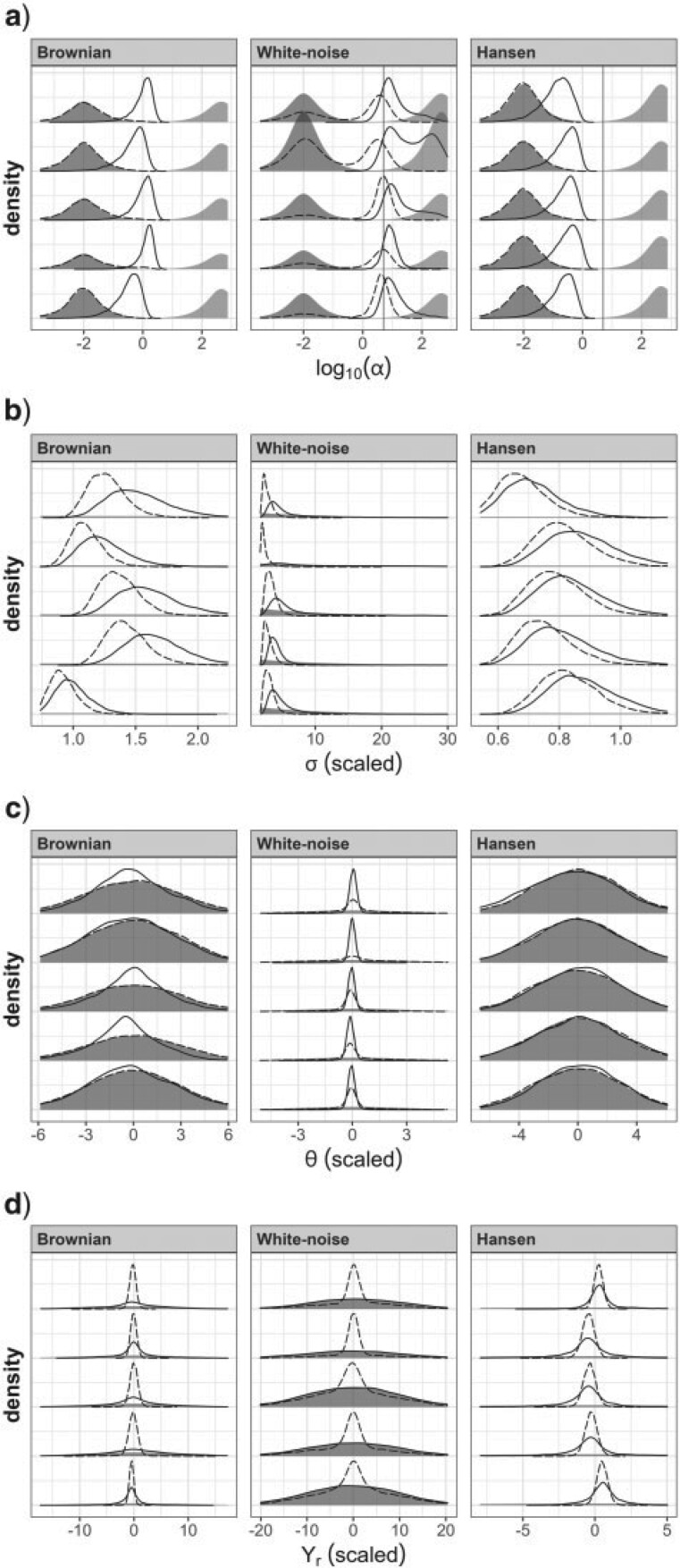
Marginal posteriors for trees with 40 tips when fitting an OU model. Dashed curves, posteriors with the BM prior. Solid curves, posteriors with the WN prior. Shaded areas represent the priors. For $\alpha$, the darker and lighter areas represent the BM and WN priors, respectively, and the vertical line represents the value used in simulations. The posterior densities for five of the 20 analyses are represented (see Appendix 8 of the Supplementary material available on Dryad for graphs for all analyses). Different columns correspond to different simulation models. The rows correspond to a) $\alpha$, b) $\sigma$, c) $\theta$, and d) $Y_{r}$. Note that because the analyses were carried out on scaled trait values, both the priors and the true values cannot be represented on the same graph for parameters in unit of traits (see Appendix 5 of the Supplementary material available on Dryad). The scaled version of such parameters is plotted here for comparison with priors.

Figure 4a shows that using one or the other prior for $\alpha$ has an influence on the marginal posterior of this parameter. For BM data, the posterior of $\alpha$ is equal to the prior when using the BM prior, as the data do not disagree with the prior. In contrast, with the WN prior, the BM data have dragged the posterior of $\alpha$ towards more Brownian values. With WN data, the marginal posterior obtained with the BM prior is bimodal, with one mode located around the true value $\alpha=5$ and a smaller mode located as the prior. This is indicative of a conflict between the prior and the data. A slight bimodality is also sometimes observed with the WN prior, with one mode located around the true value, and a smaller mode located as the prior. For Hansen data, the phylogenetic signal produced by the two clades having different selective optima is interpreted as evidence for low selection. Consequently, the BM prior is not contradicted by the data, producing a posterior identical to the prior, while the posterior obtained with the WN prior is located under much more Brownian values of $\alpha$ than the prior. 

In summary, the chosen prior had an influence on the estimated value of $\alpha$, for all types of data sets. This provides ground for the marginal posteriors of the other parameters to be influenced by the prior for $\alpha$, through the interactions among the parameters outlined above. 

**Figure 5:**
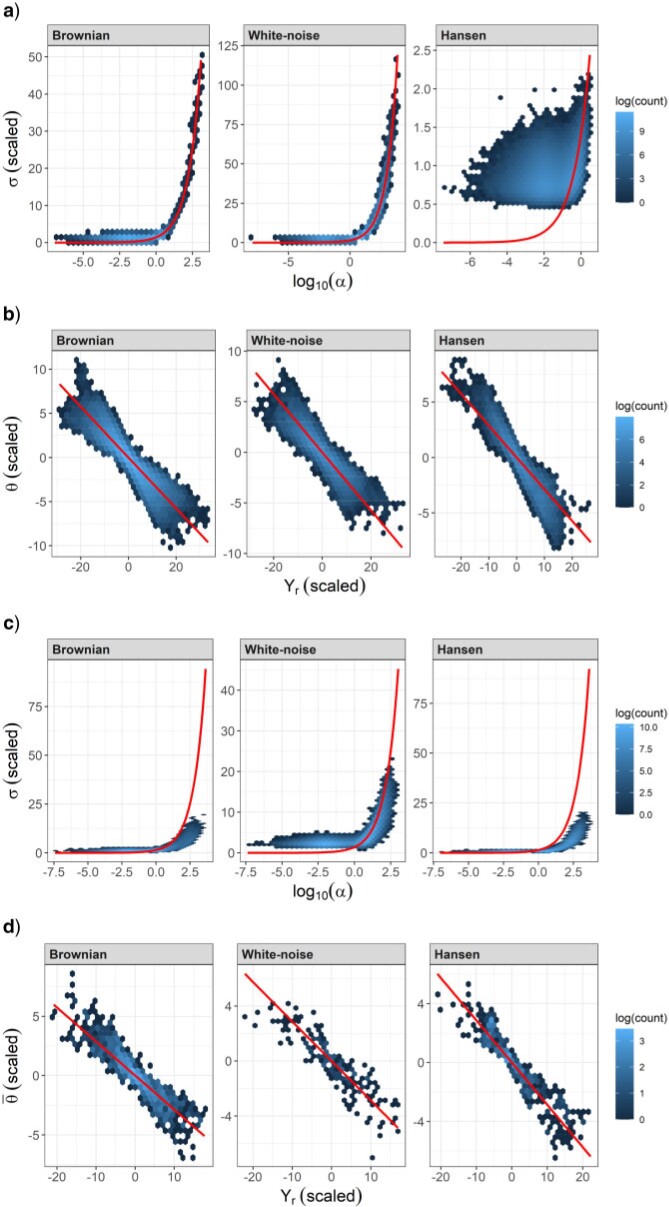
Correlation among parameters in the joint posterior. Each plot was drawn by pooling the posterior samples of all analyses conducted on data sets that were produced with the same simulation model. a) and c) Correlation of $\alpha$ and $\sigma$ for inference with the OU and Hansen models, respectively. The curve $\eta=\sigma^{2}/2\alpha=1$ is represented. b) Correlation of $\theta$ and $Y_{r}$ for inference with the OU model. Only posterior samples with intermediate $\alpha$ values (i.e., between 1 and 2) were included. The curve $\theta\left(1-e^{-1.5}\right)+Y_{r}e^{-1.5}=0$ is represented. d) Correlation of $Y_{r}$ and $\bar{\theta}$ for inference with the OU model. $\bar{\theta}$ is the average of $\theta$ across branches. Only posterior samples with intermediate $\alpha$ values (i.e., between 1 and 2) were included. The curve $\bar{\theta}\left(1-e^{-1.5}\right)+Y_{r}e^{-1.5}=0$ is represented. Note that in the Hansen model, the formula of the expected relationship between $\bar{\theta}$ and $Y_{r}$ is not this curve, although in this case it comes close to it.

For BM and Hansen data, the marginal posterior distribution of $\sigma$ is located in a region wherein the prior appears flat, indicating that in these cases estimates of $\sigma$ are likely driven by the data rather than by the prior. This was however not necessarily the case for WN data, for which we notice that the prior is more curved in the range occupied by the posterior (Fig. 4b). This should draw our attention, as it is possible that the prior for $\sigma$ prevents the posterior from moving towards higher values. Furthermore, Figure 5a shows that the values of $\alpha$ and $\sigma$ are highly positively correlated when $\rho$ is roughly higher than 0.65 (i.e., $\log_{10}(\alpha)\gtrsim 0$), which is the manifestation of the weak identifiability of these parameters outlined above. In this context, it is possible that the prior for $\sigma$, by excluding higher values, prevents $\alpha$ from reaching higher values. The analyses shown in Appendix 7 of the Supplementary material available on Dryad with a flatter prior for $\sigma$ indicate that this happened for a number of analyses: with the WN data and the WN prior, the posterior of $\sigma$ has a fatter tail, and the upper posterior mode for $\alpha$, located close to the prior, becomes dominant. 

As predicted above and illustrated in Figure 2, the posterior of $\theta$ is similar to the prior whenever the posterior of $\alpha$ is BM-like, while it is concentrated around 0, the sample mean of tip trait values, when $\alpha$ is estimated to be higher (Fig. 4c). The inverse is true for the posterior of $Y_{r}$, which is estimated close to 0 when $\alpha$ is low, and similar to its prior when $\alpha$ is high (Fig. 4d). This situation extends to all ancestral trait values besides $Y_{r}$, as shown in Appendix 8 of the Supplementary material available on Dryad. 

Appendix 7 of the Supplementary material available on Dryad shows that the influence of the prior for $\alpha$ is lesser with trees of 300 tips and for WN data sets. As these data sets are more informative than trees with 40 tips, the true value is recovered regardless of the prior for WN data. The estimates of the other parameters also became less dependent on the prior, but the correlations among parameters illustrated in Figure 5 due to nonidentifiability largely remain. For the trees simulated under the birth–death model, the results are overall similar to pure-birth trees, although we note that, for WN data and the BM prior, the upper mode located around the true value (in Fig. 4a) becomes much smaller. With the WN prior, the upper mode located around the prior altogether disappears. Both these effects are likely due to the fact that birth–death trees, through their having younger nodes, have favored the production of trait data sets with more phylogenetic structure, which is interpreted by the model as evidence for more BM processes. 

### 6.2. Hansen Model

**Figure 6:**
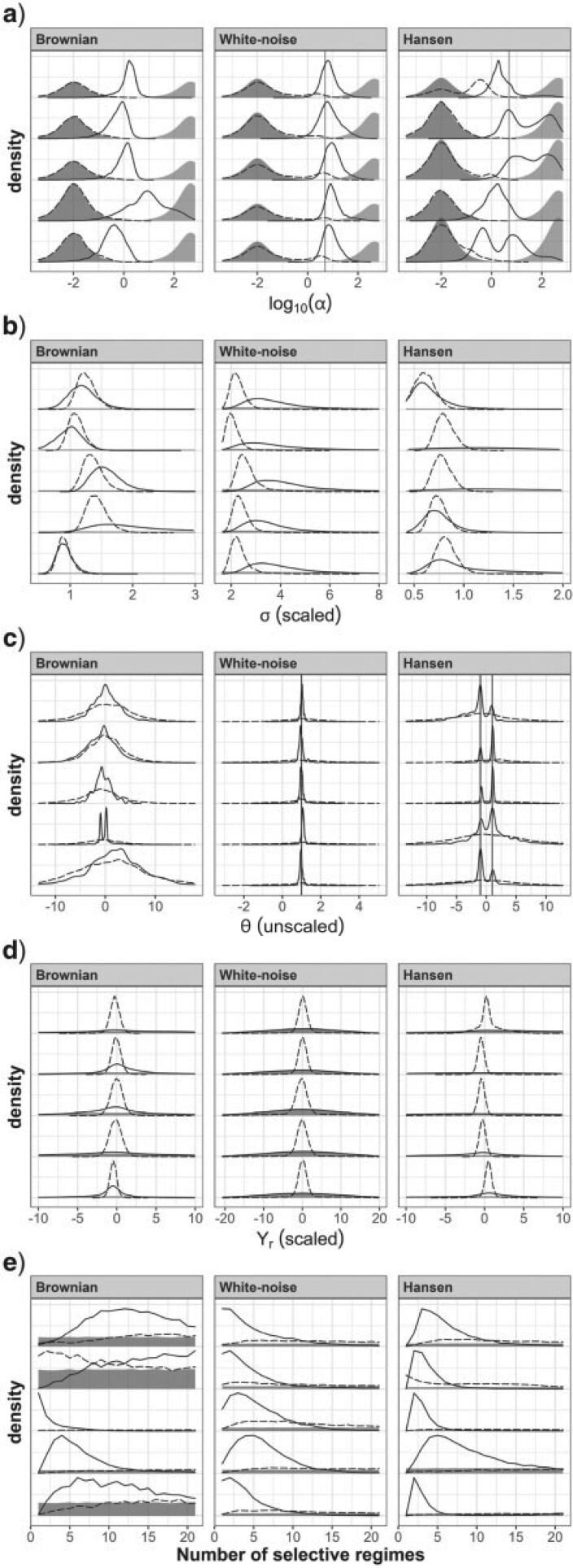
Marginal posteriors for trees with 40 tips when fitting the Hansen model with multiple selective optima. The dashed and solid curves represent the posteriors obtained with the BM and WN priors, respectively. Shaded areas represent the priors. For $\alpha$, the darker and lighter shaded areas represent the Brownian and WN priors, respectively. For $\alpha$ and $\theta$, the vertical line(s) represent(s) the value(s) used in simulations. The posterior densities of 5 of the 20 analyses are represented. Different columns correspond to different simulation models. The rows correspond to: a) $\alpha$, b) $\sigma$, c) the $\theta$’s (pooled together), d) $Y_{r}$, and e) $m$ (the number of selective regimes). The plots of e) are represented for $m\leq20$ for visibility, but the prior is flat from 0 to the number of branches (see Appendix 3 of the Supplementary material available on Dryad). The scaled version of parameters is plotted here for comparison with the priors, except for the $\theta$’s which have been unscaled for comparison with the true values.

The analyses with the Hansen model yielded results that were overall similar to analyses with the OU model (Fig. 6). In particular, the prior for $\alpha$ had a strong influence on the results, with larger posterior values of $\alpha$ with the WN prior (Fig. 6a). The main difference between the two sets of analyses was logically observed with the Hansen data sets. While the OU model accommodated the difference in trait values between the two clades with different regimes in Hansen data sets by invoking low values of $\alpha$, the Hansen model yielded larger posterior values of $\alpha$, especially with the WN prior. Such high values of $\alpha$ are indeed more likely under the Hansen model than under the OU model, because the former can accommodate the observed phylogenetic structure of trait values by invoking different selection regimes. This is obvious when looking at the posterior distribution of the selective optima obtained for Hansen data, which has two modes located around the two true values of $\theta$ with the WN prior (Fig. 6c). 

The interactions between parameters when fitting the Hansen model are also similar to what was observed with the OU model. In particular, the posterior of $Y_{r}$ is narrow and those of the $\theta$’s are wide whenever $\alpha$ is estimated to below, and conversely when $\alpha$ is estimated to be high, implying that the effect of the nonidentifiability of $Y_r$ and the $\theta$’s remains with the Hansen model (Fig. 6). This lack of identifiability is further exemplified by the same negative correlation between the values of the $\theta$’s and $Y_{r}$ (Fig. 5d). Finally, we note the same correlation between $\sigma$ and $\alpha$ when $\alpha$ is high, with the difference that the relationship between $\sigma$ and $\alpha$ is no longer the curve $\eta=1$ (Fig. 5c). This is expected as, in the Hansen model with strong selection, the sample variance of tip trait values (equal to 1) is expected to be made up of the variance of tip trait values around their mean ($\eta$), plus the variation of the $\theta$’s among selective regimes, so that $\eta$ is expected to be less than 1. 

With the Hansen model, the prior for $\alpha$ further impacts the number of selective regimes ($m$, Fig. 6e). With the BM prior, we see that the posterior of $m$ is rather flat (as is the prior for $m$). The standard Bayesian interpretation of observing a posterior close to the prior is that the data are not very informative about the parameter. However, in the present case, this conclusion is conditional on the estimate of $\alpha$. Indeed, whenever $\alpha$ is low (as was the case here with the BM prior), the influence of selection is so low that the value of $m$ no longer impacts the posterior significantly. We thus observe the posterior of $m$ close to the prior, not because the data are uninformative, but because our choice of prior restricted inference to BM-like models. Another important aspect to consider when interpreting the estimate of $m$ is the quantitative difference in the values of the $\theta$’s for the different regimes. For instance, in Figure 6e, inspecting the posterior of $m$ for Hansen datasets and the WN prior does not generally show evidence in favor of $m=2$, the true value. However, upon inspection of the posterior distributions of the $\theta$’s in Figure 6c, it is clear that whenever $m>2$, the $m$ different values of $\theta$ fall into only two significantly different modes. This suggests that there are only two significantly different selective regimes, which is the correct answer in this case. 

## Summary on Parameter Estimation with the OU and Hansen Models

The previous sections have shown that choosing a prior for $\alpha$ is not so straightforward and that it is easy to set a prior for $\alpha$ that is very stringent in favor of BM or WN models. Translating the $\alpha$ prior on the scale of $\rho$ allows to have a clear sight of whether the prior favors such extreme models. If this is the case, and data are not informative enough to contradict the prior, inference may in effect be carried out with a BM or WN model, which may have consequences on the estimation of all the other parameters ($\theta$, $Y_r$, and $\sigma$). 

In particular, leaning on the BM side will likely induce a narrow posterior for $Y_r$ as compared to the prior, which is actually only indicative of the fact that the slope of the $\theta-Y_r$ ridge is highly negative (as in Fig. 2a). In this case, conclusions about $Y_r$ must be done conditional on the prior for $\theta$ and are only valid if the low estimate of $\alpha$ is correct and not caused by a poor prior choice. The converse happens when one leans on the WN side, with a narrow posterior for $\theta$ being the reflection of the prior for $Y_r$ (as in Fig. 2b). The OU model shows here a rather counter-intuitive behavior, and I hope that this study can make this clearer. Most importantly, investigators must be aware that there is no way to estimate either $\theta$ or $Y_r$ with contemporaneous data, without prior information about one of these parameters, as already emphasized in other studies (see Ho and Ané 2014). 

We have also seen that the parameters $\alpha$ and $\sigma$ are weakly identifiable whenever $\alpha$ and $t_H$ translate into a high-enough value of $\rho$. This may induce a strong correlation of the estimates of $\alpha$ and $\sigma$, which provides the opportunity for the prior of one of these parameters to impact the posterior of the other, as shown in Figure 2c,d. The priors for these two parameters may even be in conflict if they intersect away from the top of the likelihood ridge. 

All these parameters are common to all types of inference with the OU model, whatever the purpose of the analysis, and the issues outlined here probably concern most types of OU analyses. For instance, if one wants to estimate ancestral trait values (e.g., Martins and Hansen 1997; Bokma 2002; Paradis et al. 2004; Harmon et al. 2008; Lemey et al. 2010; Revell 2012; Landis et al. 2013; Elliot and Mooers 2014; Kratsch and McHardy 2014; Meseguer et al. 2018), the estimates are conditional on the prior for $\theta$ and the estimate of $\alpha$ (Appendix 9 of the Supplementary material available on Dryad shows that the $\theta-Y_r$ ridge extends to the trait values of all ancestral species). If one wants to estimate the rate of phenotypic evolution $\sigma$, or its variation among clades (e.g., O’Meara et al. 2006; Revell and Harmon 2008; Revell and Collar 2009; Eastman et al. 2011; Jones et al. 2015; Martin 2016), the interaction between the priors for $\alpha$ and $\sigma$ may impact the results significantly. If the investigator wants to test whether selection has had a significant impact on the evolution of traits, by comparing a BM model with an OU model (e.g., Butler and King 2004; Collar et al. 2009; Harmon et al. 2010; Beaulieu et al. 2012; Cooper et al. 2016), the prior for $\alpha$ may entirely determine the results. In the case that we want to determine if the data are consistent with a Hansen model with several selective regimes (e.g., Uyeda and Harmon 2014; Cuff et al. 2015; Vining and Nunn 2016; Uyeda et al. 2017), obtaining a proper estimate of $\alpha$ is necessary. Studies that test for the impact of a predictor trait on a response trait (e.g., Davis et al. 2012; Gohli and Voje 2016; Solbakken et al. 2017; Lattenkamp et al. 2021) are also concerned. In particular, we note that the $\rho$ coefficient of the SLOUCH model (Hansen et al. 2008), which takes value 1 when the effect of selection is maximal, and value 0 when it is minimal, corresponds to $\rho(t_H/2)$ of the present study. It appears that $\rho(t_H)$ and $\rho(t_H/2)$ are very similar metrics, so that the extreme $\alpha$ priors studied in the present article translate into stringent priors for both $\rho(t_H)$ and $\rho(t_H/2)$. Hence, if SLOUCH were used in a Bayesian context with, for instance, the BM prior for $\alpha$ of Martin (2016), the analysis would almost deterministically conclude that phylogenetic inertia, rather than selection, affected the response variable. Note that most of the literature cited here resorted to maximum-likelihood inference, which does not use priors. However, the advent of Bayesian programs may soon allow these models to be implemented for Bayesian inference (most of them could already be implemented in RevBayes). 

## Interpretation of $\alpha$ or $\rho$

The speed at which trait values are attracted towards the optimum, in units of $\text{traits}/\text{time}$, is given by $\frac{1}{\alpha}(\theta - Y(t))$. The inverse of $\alpha$ thus acts as a coefficient modulating the speed of attraction by selection. We hardly have any experience in real life of such a quantity as $\alpha$, in unit of $\text{time}^{-1}$, and it is certainly not common to find estimates of selection in the biological literature in this unit. As a consequence, it may be preferable to find a transformation of $\alpha$ whose meaning is clearer. 

The meaning of the phylogenetic half-time ($t_{1/2}=\ln 2 / \alpha$) is much clearer: it is the time necessary for a trait value to cover half the way to the optimum, whatever the starting point. An evolutionary biologist certainly has a better intuition of whether, for instance, a hundred thousand years for a trait value to climb half way up a hill in an adaptive landscape is plausible or not in their study system. 

I find that the quantity $\rho$ is another meaningful transform of $\alpha$ (and $t_H$), which renders the cumulative effect that selection has had on the traits, from the root to the tips of the particular tree being studied. It is a direct reflection of the shape of evolutionary trajectories, which should be quite meaningful to many investigators, and the same value of $\rho$ represents the same trajectory shape in every system. In contrast, neither $\alpha$ nor $t_{1/2}$ bear such a universal meaning, as the same value of any one of these quantities can translate into flat or steep trajectories, depending on the height of the tree at study. In fact, it is common place for investigators using the OU model to interpret $\alpha$ or $t_{1/2}$ relative to tree height (e.g., Hansen et al. 2008; Ané et al. 2017; Cooper et al. 2016). 

In the shoes of an investigator that has no prejudice on whether selection has been influential in determining the trait values under study, parameterizing the OU model in terms of $\rho$ rather than $\alpha$ or $t_{1/2}$ thus makes sense to me. The investigator can choose a prior distribution for $\rho$ and verify graphically how this prior translates in terms of expected trajectories. It can be verified that the prior blends flat (BM-like) and steep (WN-like) trajectories, thus expressing correctly an a priori uncertainty about the relevance of selection in the study system. Also notice that many studies have used the OU model as a means for testing whether selection has had an effect at all, through the statistical comparison of a BM versus an OU model (e.g., Butler and King 2004; Collar et al. 2009; Harmon et al. 2010; Beaulieu et al. 2012; Cooper et al. 2016), concluding that selection had a significant effect when the OU model is better than the BM model. However, as shown in Appendix 2 of the Supplementary material available on Dryad, the value of $\rho$ determines how close the OU model is to a BM model independently from tree height, while $\alpha$ does not. Thus, setting a prior on $\alpha$ that favors extreme $\rho$ values may have a substantial impact on model selection. For instance, if the true model is an intermediate OU model with, say, $\rho=0.3$, and the $\alpha$ prior is WN (such as the prior of Uyeda et al. 2017), the comparison is actually made between BM and WN, and the rejection of the WN model is incorrectly interpreted as a rejection of the OU model. In contrast, a prior for $\rho$ that includes low and high values would not favor one or the other model a priori. 

In opposition to the use of $\rho$ instead of $\alpha$ or $t_{1/2}$ is the following argument. We note that $\rho$ is not a parameter *sensu stricto*, as it is calculated from $\alpha$ (a parameter) and $t_H$ (a datum). Consider now an investigator who samples a natural process at time $t_{H1}$ and assigns some prior for $\rho$, which translates into some prior for $\alpha$. Had this investigator sampled the process at a later time $t_{H2}$, by setting the same prior for $\rho$, they would have assumed a different prior for $\alpha$. This means that the a priori of the investigator about the absolute magnitude of selection ($\alpha$) in one given natural system depends on the time of sampling. One could rightfully argue that the same prior should be assigned for the same natural process, independently of the time at which the process is observed. Note that the same argument would preclude scaling the tree to unit height, as recommended in other studies (e.g., Cooper et al. 2016). While I consider this argument to be valid, I think that, in practice, investigators that are unsure about how to formulate a prior about $\alpha$ or $t_{1/2}$ in their original unit, had better set the prior on $\rho$, and check the shape of evolutionary trajectories that the prior corresponds to. In fact, as we will see below, a uniform prior on $\rho$ is able to correctly estimate $\alpha$, for data sets simulated under BM or WN. 

Finally, notice that, although I focused here on $\rho(t_H)$, others may prefer to calculate $\rho$ for another reference time period. For instance, considering that most of the tree nodes are located closer to the present, using $\rho(t_H/2)$ may seem more meaningful to some. Also, if one knows approximately the generation time $g$ of the studied organisms, the metric $\rho(g)$ reflects the percent variance that is lost in one generation due to selection. 

## Reparameterization of the OU Model

When using models like the OU model, whose parameters are not separately identifiable, or weakly so, Bayesian inference provides a sensible means to leverage prior information to identify the parameters. Investigators that have such prior knowledge about some parameters should implement it, by expressing a prior on a scale that they feel comfortable interpreting. Therefore, this study should not be interpreted as an argument against setting a prior on $\alpha$ or $t_{1/2}$ in time units, but rather addresses investigators that have vague or no prior information about the OU parameters. I particularly want to emphasize the fact that a prior that may seem uninformative on a certain scale, may appear very stringent when looked at on another scale. Furthermore, the prior for one parameter (especially $\alpha$) may have cascading effects on the other parameters, in ways that may not be immediately obvious. 

These words of caution should not be taken to mean that the OU model cannot be used when one has no prior information, and I propose here a reparameterization of the OU model, building on that of Ho and Ané (2014), that seems to behave nicely all along the BM–WN continuum. 

The OU model is reparameterized in terms of parameters that are fully identifiable, and for which I think we can easily set a meaningful prior distribution. These parameters are $\rho$, the expected tip trait value $\beta$ (see Ho and Ané 2014), and the variance of tip traits $V$. Appendix 10 of the Supplementary material available on Dryad shows how these three parameters are sufficient information for calculating the OU likelihood, and how the parameters $\alpha$ and $\sigma$ can be deduced from these new parameters. Given the nonidentifiability of $\theta$ and $Y_r$, it is necessary to further assume a value for $\theta$ to deduce a value for $Y_r$, or vice versa. But, this is only necessary for those who are specifically interested in these parameters. 

We have seen above the meaning of $\rho$, and a uniform prior distribution for $\rho$ between 0 and 1 blends trait trajectories ranging from flat (BM) to steep (WN), expressing our prior ignorance about the net effect of selection. Some investigators may prefer using a beta prior for $\rho$ and play around with the parameters to adjust the prior mix of trait trajectories as they see fit. 

Parameter $\beta$ should be estimated to lie somewhere around the sample mean $\mu$ of tip trait values. It is thus logical to choose a prior for $\beta$ that is centered around $\mu$. A normal distribution with mean $\mu$ and a standard deviation equal to two times the sample standard deviation should do nicely, allowing the estimate of $\beta$ to deviate substantially from $\mu$. 

For high values of $\rho$, covariances of trait values among species are close to 0, so that $V$ should be estimated close to the sample variance $v$ of tip traits. For low values of $\rho$, due to higher covariances, $V$ is expected to be greater than $v$. Appendix 10 of the Supplementary material available on Dryad shows that values of $V$ higher than $3v$ are highly unlikely. A log-normal prior for $V$ with a mode at $v$ and a standard deviation on the log scale equal to 0.5 (which has most of the density between 0 and $3v$) should thus be appropriate. 

**Figure 7:**
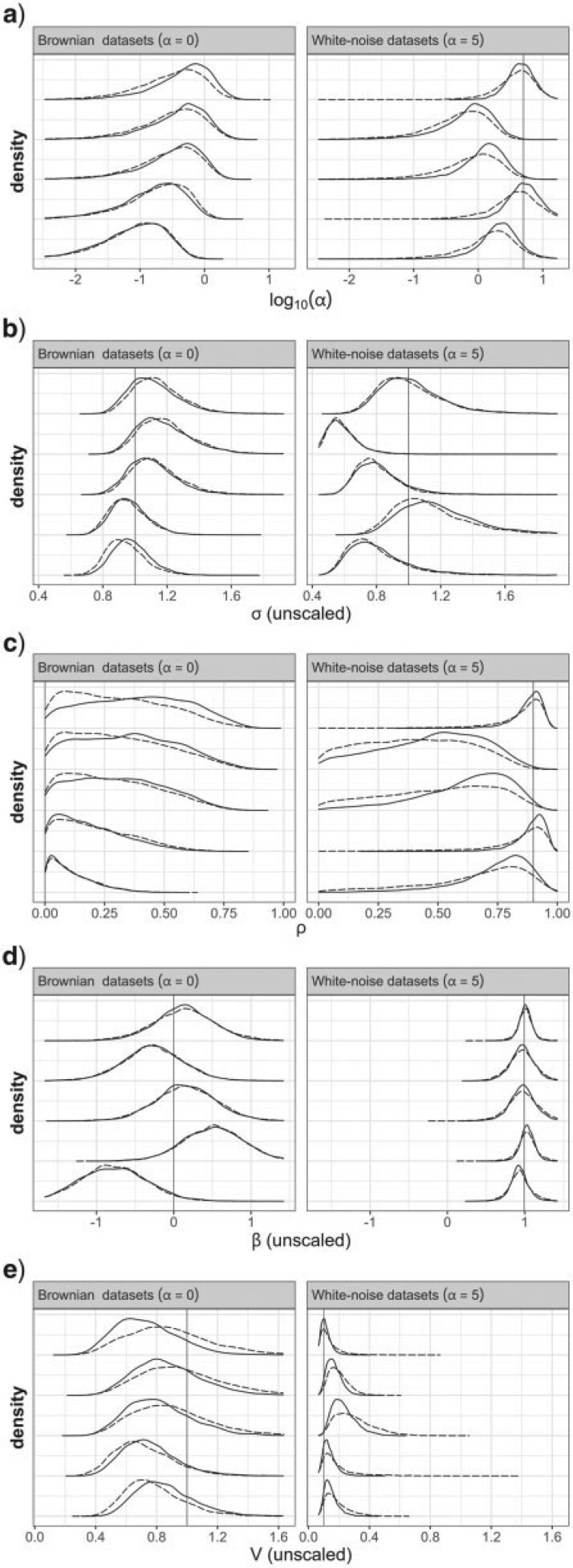
Marginal posterior densities obtained with the reparameterized OU model. a) Posterior of $\alpha$, directly comparable to Figure 4a. b) Posterior of $\sigma$, comparable to Figure 4b, except here $\sigma$ is unscaled for comparison with the true value. c) Posterior of $\rho$. d) Posterior of $\beta$ (unscaled). e) Posterior of $V$ (unscaled). The data sets used here are a subset of those used for Figure 4: 5 BM ($\alpha=0$) and 5 WN ($\alpha=5$) data sets. The vertical lines show the true values of the parameters. The prior for $\rho$ was a uniform distribution. The prior for $\beta$ was a normal distribution centered around the sample mean of tip traits and SD of 2. Two priors were considered for $V$, consisting of log-normal distributions with a mode at the sample variance of tip traits, and with an SD on the log scale of 0.5 (solid curves) or 2 (dashed curves). $\alpha$ and $\sigma$ are not parameters of the reparameterized OU model, and their values were deduced a posteriori from the values of $\rho$, $\beta,$ and $V$ (see Appendix 10 of the Supplementary material available on Dryad).

To assess the behavior of this reparameterization, it was applied to the same simulated data sets as above (simulated under BM or WN, with a single selective optimum). Figure 7 shows an overall good coverage, for both BM- or WN-simulated data sets, suggesting that the reparameterization is a sensible way to use the OU model in the absence of prior information. 

Appendix 11 of the Supplementary material available on Dryad provides a RevBayes script to implement this reparameterization, fully or partially: investigators can choose between setting a prior on $\rho$, $\alpha,$ or $t_{1/2}$; on $\beta$ alone or on any combination of two parameters among $Y_r$, $\theta,$ and $\beta$; on $V$, $\sigma,$ or $\eta$ (the stationary variance). Notice that, based on Ho and Ané (2014), I suspect that the same type of reparameterization could be used for Hansen’s model for regimes applying to connected subtrees, albeit with several values of $\beta$ for the different regimes. 

## Conclusion

Bayesian inference with the OU model may behave in an unexpected manner due to complex interactions among parameters, caused by ridges in the likelihood function. In this context, investigators should be aware that some of their conclusions are highly dependent on the chosen priors. In particular, the value of the parameter $\rho$ (related to the parameter $\alpha$ for the strength of selection and to tree height), which conveys a sense of the relevance of selection in determining trait values, largely determines the outcome of inference, whatever its purpose (i.e., estimate the strength of selection, ancestral traits, the neutral rate of evolution, compare models with or without selection, assess the impact of a trait on another, etc...). I hope that this article can entice investigators to cautiously consider the priors they use when using the OU model. I think that despite these potential pitfalls, investigators who have little prior information about the parameters should not be discouraged to carry out OU inference, for even in this situation comparative data can still provide useful insights into trait evolution. It seems that the proposed reparameterization of the OU model may help achieve this, or at least be a good tool for safety check. 

## Supplementary material

Data available from the Dryad Digital Repository: https://doi.org/10.5061/dryad.vdncjsxrc.
